# Supplementary material for: Bifidobacterium adolescentis Is Effective in Relieving Type 2 Diabetes and May Be Related to Its Dominant Core Genome and Gut Microbiota Modulation Capacity
Source: Nutrients. 2022 Jun 15;14(12):2479. doi: 10.3390/nu14122479 (PMC9227778; doi:10.3390/nu14122479)
Supplement: Supplementary file 1 [file nutrients-14-02479-s001.zip › nutrients-1765916-supplementary.pdf]

Bifidobacterium adolescentis is effective in relieving type 2 diabetes and may be related to its dominant core genome and gut microbiota modulation capacity

Xin Qian[1, 2, #], Qian Si[1, 2, #], Guopeng Lin[1, 2], Minmin Zhu[3]*, Jingyu Lu[1, 2, 4], Hao Zhang[1, 2, 4, 5], Gang Wang[1, 2, 5]*, Wei Chen[1, 2, 4]

1. State Key Laboratory of Food Science and Technology, Jiangnan University, Wuxi, Jiangsu 214122, China.
2. School of Food Science and Technology, Jiangnan University, Wuxi, Jiangsu 214122, China.
3. Department of Anesthesiology, Wuxi No. 2 People's Hospital, Wuxi, Jiangsu 214002, China.
4. National Engineering Research Center for Functional Food, Jiangnan University, Wuxi, Jiangsu 214122, China.
5. (Yangzhou) Institute of Food Biotechnology, Jiangnan University, Yangzhou 225004, China.

[#] These authors contributed equally to this work.

Corresponding author:

*Minmin Zhu

E-mail: mmzhummzhu@163.com

*Gang Wang,

E-mail: wanggang@jiangnan.edu.cn,

Phone: (86)510-85912155

1 Supplementary Methods

1.1 Feed and gavage formulations

Low-fat diet: energy: 3.6 kcal g^{-1}; 10% kcal from fat, 71% kcal from carbohydrate, and 19% kcal from protein. High-fat diet: energy: 5.1 kcal g^{-1}; 60% kcal from fat, 21% kcal from carbohydrate, and 19% kcal from protein. Gavage formulation of Control or Model group: 0.2 mL 3% sucrose solution. Gavage formulation of Metformin group: 200 mg per kg·bw metformin dissolved in 3% sucrose solution. Gavage formulation of Treatment group:0.2 mL 2×10^8 CFU per mL bacterial suspension resuspended in 3% sucrose solution

1.2 Bioinformatical analysis of gut microbiota

Bioinformatical analysis was performed as previously described (Molecular Nutrition & Food Research, 2021, 65(8): 2000704). The raw data of sequencing was processed using Quantitative Insights into Microbial Ecology (QIIME) wrapper and software (Caporaso, J, et al. Nature methods 2010, 7, 335). The operational taxonomic unit (OTU) was picked using a criterion of 97% nucleotide identity and the taxonomy was assigned using the SILVA database. The Shannon and Chao 1 indices were measured from the rarefied OTU. β-diversity was estimated using compositional data analysis by PCA using Aitchison distance (Gloor, G. B, et al. Front. Microbiol. 2017, 8, 2224), following a PERMANOVA test to assess differences. LEfSe was used to differentiate microbial taxa (Wilcoxon rank-sum test, $\alpha < 0.05$ and log LDA>2.0 were used as the threshold). GBMs analysis was performed as previously described (Valles-Colomer, M, J, et al.

Nature microbiology 2019, 4, 623). Briefly, the metagenomic composition was predicted from 16S rRNA sequences using PICRUST2 (Nature Biotechnology, 2020, 38(6): 685-688). The identified KEGG Orthogroups (KOs) abundance was normalized and mapped to 56 GBMs using the Omixer-RPM V1.0. The GBMs enrichment differences were calculated by Welch's t-test, P<0.01.5 adjusted by the Benjamini & Hochberg method were considered significantly different.

1.3 Bioinformatical analysis of genome

Reads were assembled with SOAPdenovo. Overall, for each strain, Prodigal (v2.6.3), Glimmer (v3.02) and GeneMarkS-2 (web-server) were run on their default settings to generate sets of predicted genes. The predicted genes were annotated in public databases including the Non-redundant (NR, download in 12/31/2021), Kyoto Encyclo-paedia of Genes and Genomes (KEGG, http://www.genome.jp/kegg), Clusters of Ortholo-gous Groups (COG, http://www.ncbi.nlm.nih.gov/COG). Additionally, the eggNOG-mapper v2.1.6 (https://github.com/eggnogdb/eggnog-mapper) was also used for further annotation. Pan-Genome Analysis Pipeline was used to analyze the pan-genome and Orthologous gene families were analyzed using OrthoMCL (version v2.0.9) and OrthoFinder. The orthologous genes of all *Bifidobacterium* genomes were aligned using MAFFT v7.3.

2 Supplementary Figures and Tables

Table S1. Genome data used in this study

B. adolescentis				*B. bifidum*			
Genome ID	Accession number	Genome ID	Accession number	Genome ID	Accession number	Genome ID	Accession number
QCNCBI1	GCF_000010425.1	QCNCBI46	GCF_003856735.1	NCBI1	GCF_000155395.1	NCBI46	GCF_003465425.1
QCNCBI2	GCF_000154085.1	QCNCBI47	GCF_004167585.1	NCBI2	GCF_000164965.1	NCBI47	GCF_003466365.1
QCNCBI3	GCF_000702865.1	QCNCBI48	GCF_005845205.1	NCBI3	GCF_000165905.1	NCBI48	GCF_003466395.1
QCNCBI4	GCF_000737885.1	QCNCBI49	GCF_009832825.1	NCBI4	GCF_000265095.1	NCBI49	GCF_003466485.1
QCNCBI5	GCF_000741415.1	QCNCBI50	GCF_013249115.1	NCBI5	GCF_000273525.1	NCBI50	GCF_003467985.1
QCNCBI6	GCF_000771705.1	QCNCBI51	GCF_014524935.1	NCBI6	GCF_000299595.1	NCBI51	GCF_003468415.1
QCNCBI7	GCF_000817995.1	QCNCBI52	GCF_015547885.1	NCBI7	GCF_000300215.1	NCBI52	GCF_003470585.1
QCNCBI8	GCF_001010915.1	QCNCBI53	GCF_015548665.1	NCBI8	GCF_000466525.1	NCBI53	GCF_003470615.1
QCNCBI9	GCF_001406215.1	QCNCBI54	GCF_015548755.1	NCBI9	GCF_000741085.1	NCBI54	GCF_003471555.1
QCNCBI10	GCF_001406455.1	QCNCBI55	GCF_015548985.1	NCBI10	GCF_000771485.1	NCBI55	GCF_003471595.1
QCNCBI11	GCF_001406735.1	QCNCBI56	GCF_015549865.1	NCBI11	GCF_001020245.1	NCBI56	GCF_003472345.1
QCNCBI12	GCF_001756865.1	QCNCBI57	GCF_015552825.1	NCBI12	GCF_001020255.1	NCBI57	GCF_003473145.1
QCNCBI13	GCF_002075965.1	QCNCBI58	GCF_015553845.1	NCBI13	GCF_001020265.1	NCBI58	GCF_003573895.1
QCNCBI14	GCF_002107925.1	QCNCBI59	GCF_015553925.1	NCBI14	GCF_001020275.1	NCBI59	GCF_003790385.1
QCNCBI15	GCF_002107955.1	QCNCBI60	GCF_015553955.1	NCBI15	GCF_001020325.1	NCBI60	GCF_004167365.1
QCNCBI16	GCF_002107975.1	QCNCBI61	GCF_015554265.1	NCBI16	GCF_001020335.1	NCBI61	GCF_004799295.1
QCNCBI17	GCF_002107995.1	QCNCBI62	GCF_015555595.1	NCBI17	GCF_001020355.1	NCBI62	GCF_005844205.1
QCNCBI18	GCF_002108015.1	QCNCBI63	GCF_015557595.1	NCBI18	GCF_001020375.1	NCBI63	GCF_005844365.1
QCNCBI19	GCF_002108035.1	QCNCBI64	GCF_015557685.1	NCBI19	GCF_001020405.1	NCBI64	GCF_008868415.1
QCNCBI20	GCF_002108045.1	QCNCBI65	GCF_015558085.1	NCBI20	GCF_001020415.1	NCBI65	GCF_009075765.1
QCNCBI21	GCF_002108075.1	QCNCBI66	GCF_015558415.1	NCBI21	GCF_001025135.1	NCBI66	GCF_009075785.1
QCNCBI22	GCF_002108095.1	QCNCBI67	GCF_015558565.1	NCBI22	GCF_001281345.1	NCBI67	GCF_009077915.1
QCNCBI23	GCF_002108135.1	QCNCBI68	GCF_015558575.1	NCBI23	GCF_001405355.1	NCBI68	GCF_009077955.1
QCNCBI24	GCF_002108155.1	QCNCBI69	GCF_015558745.1	NCBI24	GCF_001546225.1	NCBI69	GCF_009077975.1
QCNCBI25	GCF_002108165.1	QCNCBI70	GCF_015559505.1	NCBI25	GCF_001595435.1	NCBI70	GCF_009078005.1
QCNCBI26	GCF_003030905.1	QCNCBI71	GCF_015560095.1	NCBI26	GCF_001685685.1	NCBI71	GCF_009078015.1
QCNCBI27	GCF_003429385.1	QCNCBI72	GCF_016069975.1	NCBI27	GCF_002076105.1	NCBI72	GCF_009078055.1
QCNCBI28	GCF_003436185.1	QCNCBI73	GCF_017815835.1	NCBI28	GCF_002114145.1	NCBI73	GCF_009078065.1
QCNCBI29	GCF_003437735.1	QCNCBI74	GCF_018785705.1	NCBI29	GCF_002845845.1	NCBI74	GCF_009078095.1
QCNCBI30	GCF_003437755.1	QCNCBI75	GCF_018785715.1	NCBI30	GCF_003094645.1	NCBI75	GCF_009078115.1
QCNCBI31	GCF_003437775.1	QCNCBI76	GCF_019041695.1	NCBI31	GCF_003094715.1	NCBI76	GCF_009159095.1
QCNCBI32	GCF_003457765.1	QCNCBI77	GCF_019041975.1	NCBI32	GCF_003094735.1	NCBI77	GCF_009159495.1
QCNCBI33	GCF_003458805.1	QCNCBI78	GCF_019042035.1	NCBI33	GCF_003094755.1	NCBI78	GCF_009159505.1
QCNCBI34	GCF_003462885.1	QCNCBI79	GCF_019042045.1	NCBI34	GCF_003094795.1	NCBI79	GCF_009159555.1
QCNCBI35	GCF_003462895.1	QCNCBI80	GCF_019127685.1	NCBI35	GCF_003095035.1	NCBI80	GCF_009160775.1
QCNCBI36	GCF_003464325.1	QCNCBI81	GCF_019127775.1	NCBI36	GCF_003390735.1	NCBI81	GCF_009160825.1
QCNCBI37	GCF_003465205.1	QCNCBI82	GCF_019127835.1	NCBI37	GCF_003436135.1	NCBI82	GCF_009160845.1
QCNCBI38	GCF_003466335.1	QCNCBI83	GCF_019128025.1	NCBI38	GCF_003436575.1	NCBI83	GCF_009160865.1
QCNCBI39	GCF_003467335.1	QCNCBI84	GCF_019129365.1	NCBI39	GCF_003436635.1	NCBI84	GCF_009160885.1
QCNCBI40	GCF_003468385.1	QCNCBI85	GCF_019131675.1	NCBI40	GCF_003436655.1	NCBI85	GCF_009160905.1
QCNCBI41	GCF_003469145.1	QCNCBI86	GCF_019734235.1	NCBI41	GCF_003437175.1	NCBI86	GCF_009160915.1
QCNCBI42	GCF_003472095.1	QCNCBI87	GCF_019734295.1	NCBI42	GCF_003437325.1	NCBI87	GCF_009160945.1
QCNCBI43	GCF_003472245.1	QCNCBI88	GCF_019972965.1	NCBI43	GCF_003437345.1	NCBI88	GCF_011029205.1
QCNCBI44	GCF_003472265.1	QCNCBI89	GCF_022133115.1	NCBI44	GCF_003437785.1	NCBI89	GCF_013867495.1
QCNCBI45	GCF_003473105.1	QCNCBI90	GCF_022135325.1	NCBI45	GCF_003437945.1	NCBI90	GCF_015549125.1

Genome ID: The ID used in this study.

Accession number: The number used to obtain genomic data in RefSeq database.

Table S2. Effects of *Bifidobacterium* on body weight and organ quality

Group	Fasting body (g)	Liver (g)	Kidney (g)
**Control**	25.47 ± 1.10^{a}	0.89 ± 0.04^{a}	0.30 ± 0.03^{a}
**Model**	25.10 ± 1.20^{a}	1.23 ± 0.09^{b}	0.33 ± 0.01^{a}
**Metformin**	25.19 ± 1.63^{a}	1.16 ± 0.15^{b}	0.34 ± 0.05^{a}
**3M10**	24.87 ± 1.11^{a}	1.06 ± 0.07^{ab}	0.32 ± 0.03^{a}
**8M4**	25.14 ± 1.39^{a}	1.02 ± 0.19^{ab}	0.33 ± 0.04^{a}
**26M1**	23.60 ± 2.18^{a}	1.01 ± 0.17^{ab}	0.31 ± 0.03^{a}
**30M5**	24.93 ± 1.33^{a}	1.14 ± 0.06^{b}	0.33 ± 0.03^{a}
**34M4**	25.25 ± 1.28^{a}	1.18 ± 0.13^{b}	0.35 ± 0.21^{a}
**41M3**	25.38 ± 1.25^{a}	1.13 ± 0.06^{b}	0.34 ± 0.03^{a}
**50M3**	25.09 ± 1.38^{a}	1.12 ± 0.07^{b}	0.33 ± 0.03^{a}
**2016**	25.41 ± 1.61^{a}	1.04 ± 0.14^{ab}	0.33 ± 0.04^{a}
**5M8**	25.69 ± 1.72^{a}	1.16 ± 0.08^{b}	0.33 ± 0.07^{a}
**7-05**	25.45 ± 0.98^{a}	1.10 ± 0.07^{b}	0.32 ± 0.03^{a}
**9M10**	26.10 ± 1.08^{a}	1.17 ± 0.07^{b}	0.35 ± 0.03^{a}
**19M10**	26.01 ± 2.12^{a}	1.19 ± 0.19^{b}	0.35 ± 0.06^{a}
**21M3**	24.28 ± 1.89^{a}	1.09 ± 0.07^{b}	0.34 ± 0.04^{a}
**23M2**	25.22 ± 2.07^{a}	1.11 ± 0.09^{b}	0.34 ± 0.04^{a}
**26M7**	25.48 ± 1.22^{a}	1.10 ± 0.09^{b}	0.34 ± 0.02^{a}
**26-7**	24.38 ± 1.22^{a}	1.13 ± 0.12^{b}	0.33 ± 0.03^{a}

Different letters a/b indicate significant differences ($P < 0.05$).

Table S3. Effect of *Bifidobacterium* on organ index

Group	Liver / body weight (%)	Kidney / body weight (%)
**Control**	3.52 ± 0.16[a]	1.19 ± 0.08[a]
**Model**	4.89 ± 0.21[c]	1.30 ± 0.04[a]
**Metformin**	4.59 ± 0.36[bc]	1.34 ± 0.12[a]
**3M10**	4.25 ± 0.24[bc]	1.29 ± 0.07[a]
**8M4**	4.08 ± 0.61[ab]	1.32 ± 0.12[a]
**26M1**	4.25 ± 0.38[bc]	1.32 ± 0.07[a]
**30M5**	4.58 ± 0.17[bc]	1.32 ± 0.06[a]
**34M4**	4.66 ± 0.38[bc]	1.37 ± 0.07[a]
**41M3**	4.44 ± 0.18[bc]	1.34 ± 0.07[a]
**50M3**	4.47 ± 0.23[bc]	1.30 ± 0.07[a]
**2016**	4.08 ± 0.44[ab]	1.29 ± 0.10[a]
**5M8**	4.50 ± 0.20[bc]	1.26 ± 0.24[a]
**7-05**	4.32 ± 0.23[bc]	1.26 ± 0.09[a]
**9M10**	4.47 ± 0.30[bc]	1.33 ± 0.07[a]
**19M10**	4.55 ± 0.48[bc]	1.34 ± 0.12[a]
**21M3**	4.51 ± 0.26[bc]	1.40 ± 0.14[a]
**23M2**	4.43 ± 0.40[bc]	1.35 ± 0.10[a]
**26M7**	4.33 ± 0.24[bc]	1.32 ± 0.05[a]
**26-7**	4.63 ± 0.31[bc]	1.29 ± 0.08[a]

Different letters a/b indicate significant differences ($P < 0.05$).

Table S4. Pathway statistics of unique core genes in two *Bifidobacterium* species

Pathway	B.ad	B.bi	Pathway	B.ad	B.bi
Glycolysis / Gluconeogenesis	2	1	**Riboflavin metabolism**	**1**	**0**
**Citrate cycle (TCA cycle)**	1	0	Vitamin B6 metabolism	1	1
**Pentose phosphate pathway**	4	0	Nicotinate and nicotinamide metabolism	2	4
**Pentose and glucuronate interconversions**	5	0	Pantothenate and CoA biosynthesis	3	4
**Fructose and mannose metabolism**	1	0	Folate biosynthesis	1	2
Galactose metabolism	2	4	**Zeatin biosynthesis**	**1**	**0**
Ascorbate and aldarate metabolism	1	3	Nitrogen metabolism	1	1
**Oxidative phosphorylation**	**2**	**0**	**Aminoacyl-tRNA biosynthesis**	**2**	**0**
**Arginine biosynthesis**	**2**	**0**	**Vancomycin resistance**	**1**	**0**
Ubiquinone and other terpenoid-quinone biosynthesis	0	1	**Cationic antimicrobial peptide (CAMP) resistance**	**1**	**0**
Purine metabolism	10	5	**Antifolate resistance**	**1**	**0**
Pyrimidine metabolism	13	3	Drug metabolism - other enzymes	0	1
Alanine, aspartate and glutamate metabolism	4	1	beta-Lactam resistance	0	2
**Glycine, serine and threonine metabolism**	**4**	**0**	ABC transporters	9	1
**Monobactam biosynthesis**	**3**	**0**	**Two-component system**	**3**	**0**
Cysteine and methionine metabolism	2	1	Quorum sensing	3	2
Valine, leucine and isoleucine biosynthesis	1	3	Biofilm formation - Escherichia coli	2	1
**Lysine biosynthesis**	**6**	**0**	Phosphotransferase system (PTS)	1	4
**Arginine and proline metabolism**	**3**	**0**	**Ribosome biogenesis in eukaryotes**	**1**	**0**
**Histidine metabolism**	**1**	**0**	Ribosome	3	1
Penicillin and cephalosporin biosynthesis	0	2	**RNA degradation**	**3**	**0**
Phenylalanine, tyrosine and tryptophan biosynthesis	1	2	**RNA polymerase**	**2**	**0**
**Taurine and hypotaurine metabolism**	**1**	**0**	**DNA replication**	**3**	**0**
**Cyanoamino acid metabolism**	**2**	**0**	**Protein export**	**2**	**0**
**D-Alanine metabolism**	**1**	**0**	**Bacterial secretion system**	**1**	**0**
**Starch and sucrose metabolism**	**4**	**0**	Base excision repair	2	1
Novobiocin biosynthesis	0	1	**Nucleotide excision repair**	**2**	**0**
Phenazine biosynthesis	0	1	**Mismatch repair**	**3**	**0**
Amino sugar and nucleotide sugar metabolism	2	7	**Homologous recombination**	**5**	**0**
**Inositol phosphate metabolism**	**1**	**0**	**HIF-1 signaling pathway**	**1**	**0**
Glycerophospholipid metabolism	3	1	**Phosphatidylinositol signaling system**	**1**	**0**
Pyruvate metabolism	3	1	**Cell cycle - Caulobacter**	**2**	**0**
**Glyoxylate and dicarboxylate metabolism**	**3**	**0**	**Peroxisome**	**1**	**0**
**Propanoate metabolism**	**3**	**0**	**Necroptosis**	**2**	**0**
Aminobenzoate degradation	0	1	**Insulin signaling pathway**	**1**	**0**
Butanoate metabolism	1	1	**Glucagon signaling pathway**	**1**	**0**
C5-Branched dibasic acid metabolism	1	1	**Insulin resistance**	**1**	**0**
**One carbon pool by folate**	**2**	**0**	**Alzheimer disease**	**1**	**0**
**Methane metabolism**	**2**	**0**	**Chagas disease (American trypanosomiasis)**	**1**	**0**
**Carbon fixation in photosynthetic organisms**	**3**	**0**	**African trypanosomiasis**	**1**	**0**
**Carbon fixation pathways in prokaryotes**	**3**	**0**	**Tuberculosis**	**1**	**0**
Thiamine metabolism	3	1	**Proteoglycans in cancer**	**1**	**0**
cGMP-PKG signaling pathway	0	1	Aldosterone-regulated sodium reabsorption	0	1
cAMP signaling pathway	0	1	Endocrine and other factor-regulated calcium reabsorption	0	1
Sulfur relay system	0	1	Proximal tubule bicarbonate reclamation	0	1
Autophagy - yeast	0	1	Salivary secretion	0	1
Cardiac muscle contraction	0	1	Gastric acid secretion	0	1
Adrenergic signaling in cardiomyocytes	0	1	Pancreatic secretion	0	1
Prolactin signaling pathway	0	1	Carbohydrate digestion and absorption	0	1
Thyroid hormone synthesis	0	1	Protein digestion and absorption	0	1
Thyroid hormone signaling pathway	0	1	Bile secretion	0	1
Aldosterone synthesis and secretion	0	1	Mineral absorption	0	1

B.ad: Number of unique core genes in *B. adolescentis*.
B.bi: Number of unique core genes in *B. bifidum*.

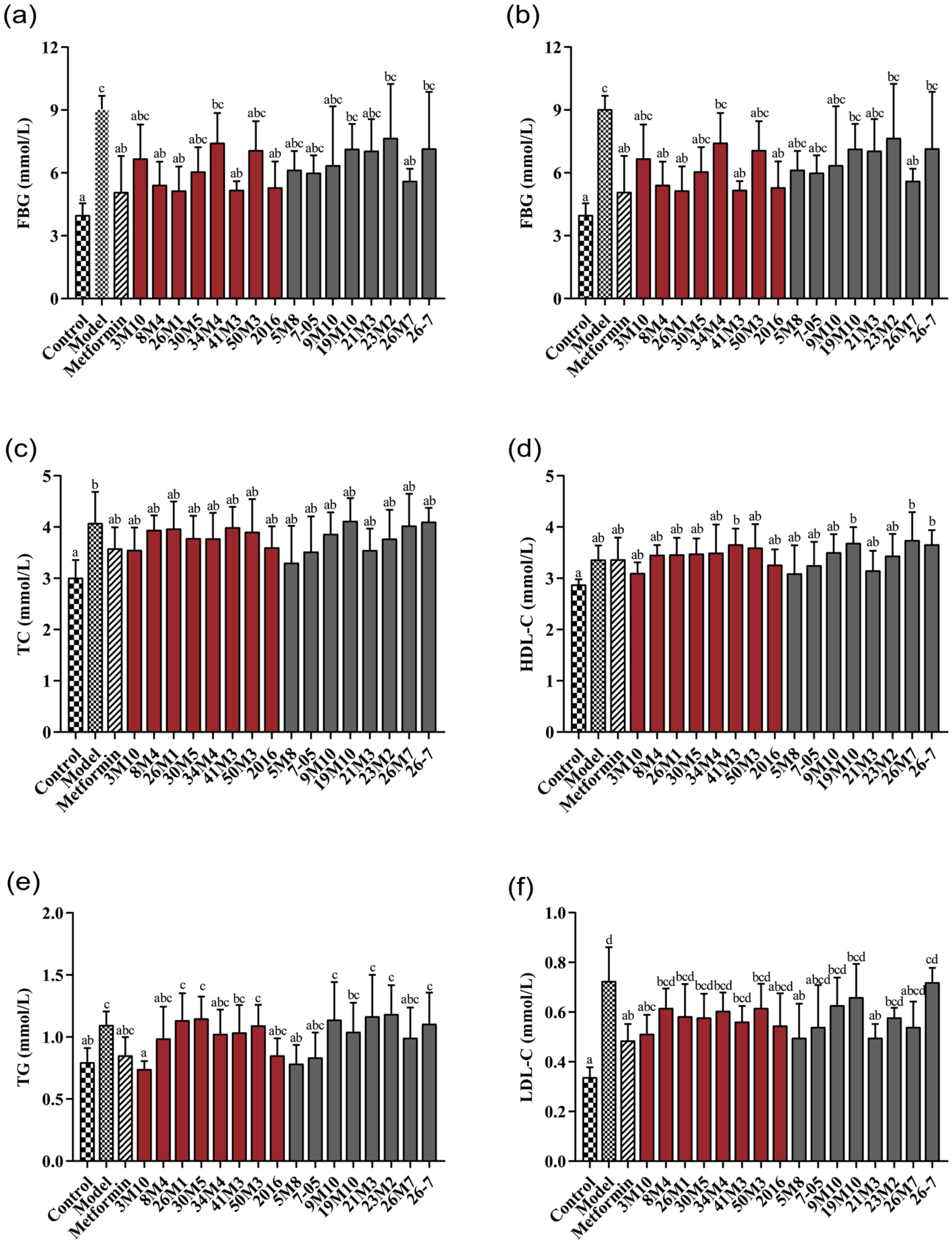

Figure S1. Effects of different *Bifidobacterium* on the regulation of glucose and lipid metabolism in T2D mice. (a, b) FBG at 1 or 5 weeks after STZ injection, respectively; (c-f) TC, TG, HDL-C and LDL-C level. Different letters represent statistically significant differences between different groups ($P < 0.05$), $n = 6$.

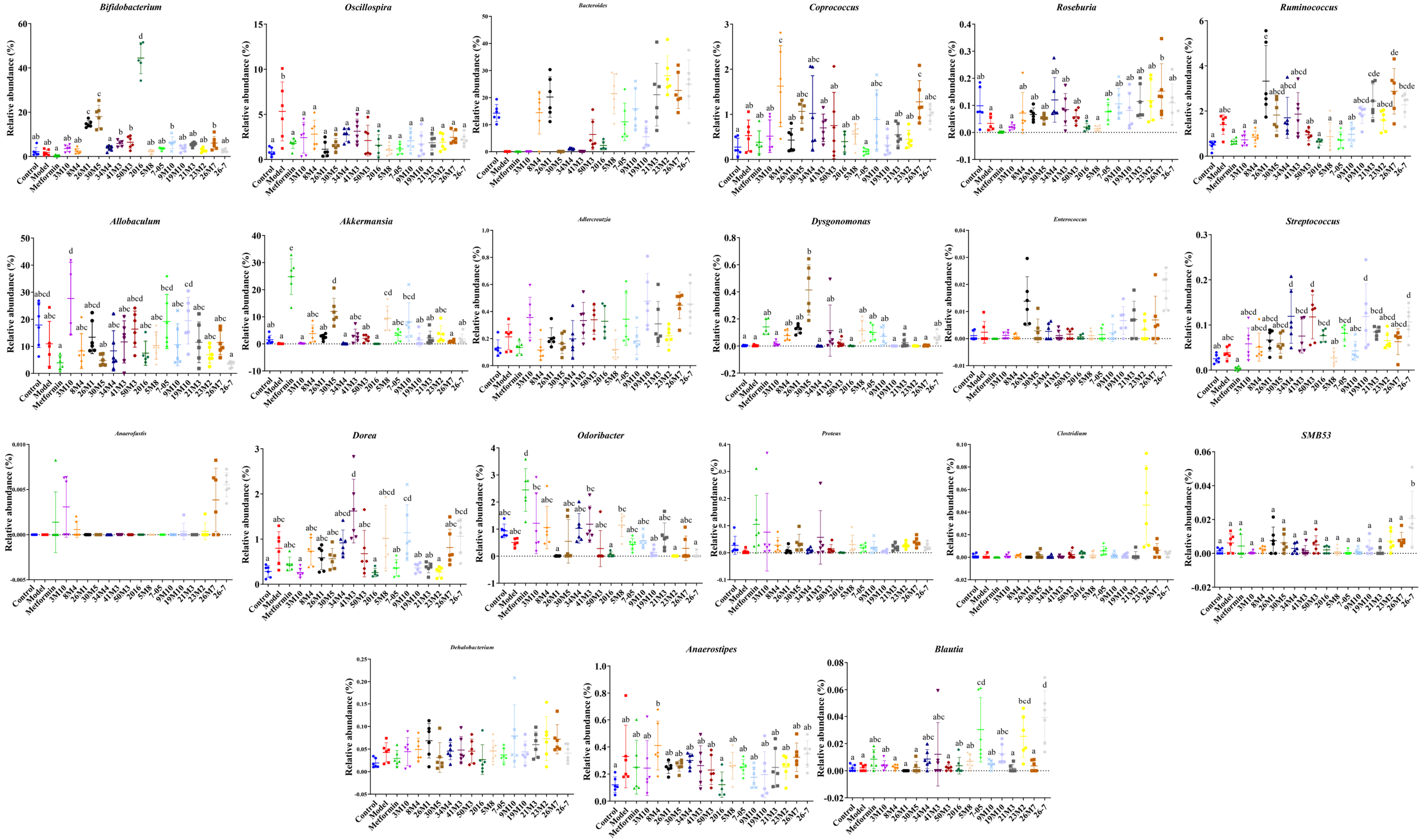

Figure S2. *Bifidobacterium* treatment alters the diversity and the structure at the species level of the gut microbiota.

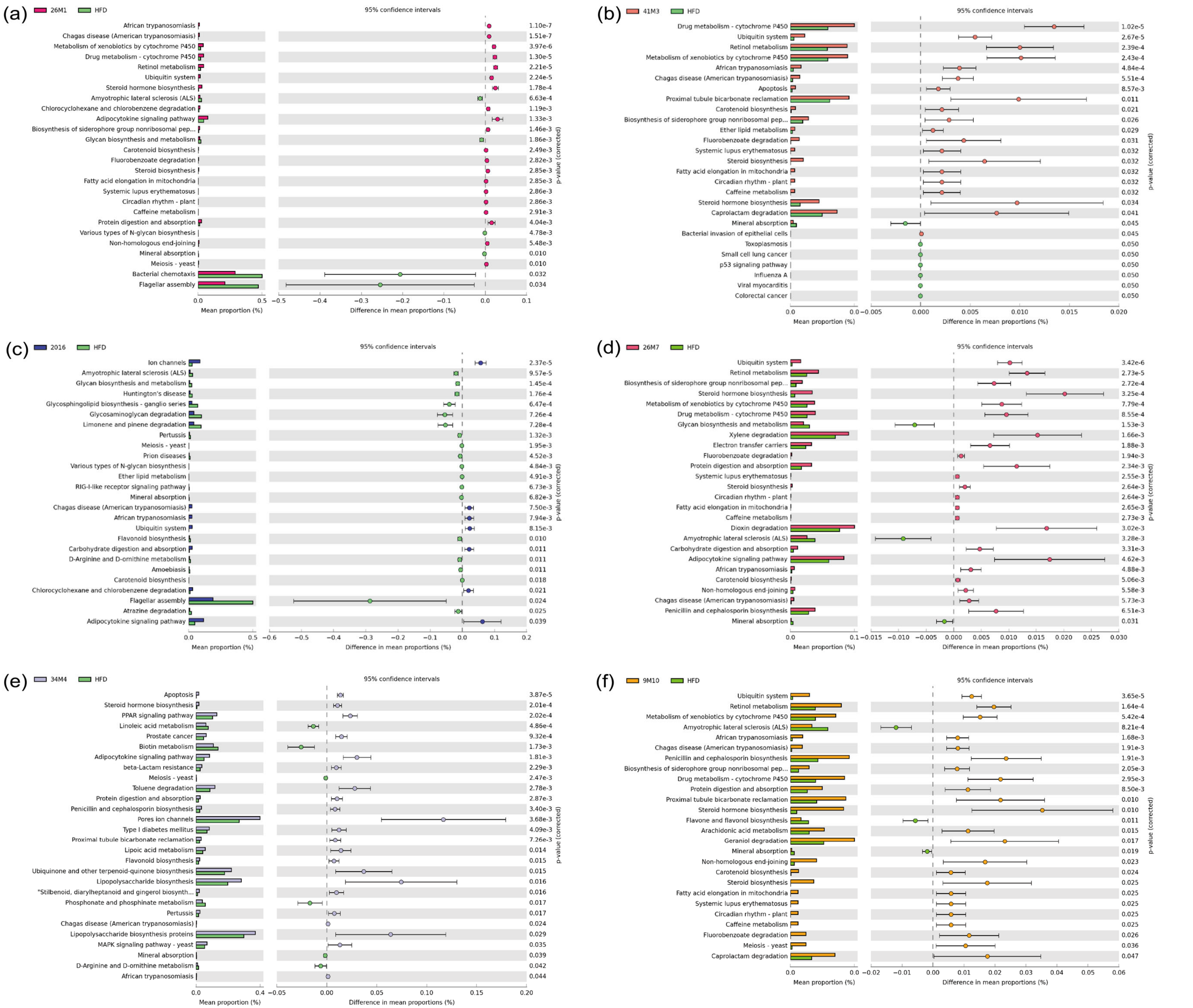
(a)
26M1
HFD
95% confidence intervals
African trypanosomiasis 1.10e-7
Chagas disease (American trypanosomiasis) 1.51e-7
Metabolism of xenobiotics by cytochrome P450 3.97e-6
Drug metabolism - cytochrome P450 1.30e-5
Retinol metabolism 2.21e-5
Ubiquitin system 2.24e-5
Steroid hormone biosynthesis 1.78e-4
Amyotrophic lateral sclerosis (ALS) 6.63e-4
Chlorocyclohexane and chlorobenzene degradation 1.19e-3
Adipocytokine signaling pathway 1.33e-3
Biosynthesis of siderophore group nonribosomal pep... 1.46e-3
Glycan biosynthesis and metabolism 1.86e-3
Carotenoid biosynthesis 2.49e-3
Fluorobenzoate degradation 2.82e-3
Steroid biosynthesis 2.85e-3
Fatty acid elongation in mitochondria 2.85e-3
Systemic lupus erythematosus 2.86e-3
Circadian rhythm - plant 2.86e-3
Caffeine metabolism 2.91e-3
Protein digestion and absorption 4.04e-3
Various types of N-glycan biosynthesis 4.78e-3
Non-homologous end-joining 5.48e-3
Mineral absorption 0.010
Meiosis - yeast 0.010
Bacterial chemotaxis 0.032
Flagellar assembly 0.034
Mean proportion (%)
Difference in mean proportions (%)
p-value (corrected)
(b)
41M3
HFD
95% confidence intervals
Drug metabolism - cytochrome P450 1.02e-5
Ubiquitin system 2.67e-5
Retinol metabolism 2.39e-4
Metabolism of xenobiotics by cytochrome P450 2.43e-4
African trypanosomiasis 4.84e-4
Chagas disease (American trypanosomiasis) 5.51e-4
Apoptosis 8.57e-3
Proximal tubule bicarbonate reclamation 0.011
Carotenoid biosynthesis 0.021
Biosynthesis of siderophore group nonribosomal pep... 0.026
Ether lipid metabolism 0.029
Fluorobenzoate degradation 0.031
Systemic lupus erythematosus 0.032
Steroid biosynthesis 0.032
Fatty acid elongation in mitochondria 0.032
Circadian rhythm - plant 0.032
Caffeine metabolism 0.032
Steroid hormone biosynthesis 0.034
Caprolactam degradation 0.041
Mineral absorption 0.045
Bacterial invasion of epithelial cells 0.045
Toxoplasmosis 0.050
Small cell lung cancer 0.050
p53 signaling pathway 0.050
Influenza A 0.050
Viral myocarditis 0.050
Colorectal cancer 0.050
Mean proportion (%)
Difference in mean proportions (%)
p-value (corrected)
(c)
2016
HFD
95% confidence intervals
Ion channels 2.37e-5
Amyotrophic lateral sclerosis (ALS) 9.57e-5
Glycan biosynthesis and metabolism 1.45e-4
Huntington's disease 1.76e-4
Glycosphingolipid biosynthesis - ganglio series 6.47e-4
Glycosaminoglycan degradation 7.26e-4
Limonene and pinene degradation 7.28e-4
Pertussis 1.32e-3
Meiosis - yeast 1.95e-3
Prion diseases 4.52e-3
Various types of N-glycan biosynthesis 4.84e-3
Ether lipid metabolism 4.91e-3
RIG-I-like receptor signaling pathway 6.73e-3
Mineral absorption 6.82e-3
Chagas disease (American trypanosomiasis) 7.50e-3
African trypanosomiasis 7.94e-3
Ubiquitin system 8.15e-3
Flavonoid biosynthesis 0.010
Carbohydrate digestion and absorption 0.011
D-Arginine and D-ornithine metabolism 0.011
Amoebiasis 0.011
Carotenoid biosynthesis 0.018
Chlorocyclohexane and chlorobenzene degradation 0.021
Flagellar assembly 0.024
Atrazine degradation 0.025
Adipocytokine signaling pathway 0.039
Mean proportion (%)
Difference in mean proportions (%)
p-value (corrected)
(d)
26M7
HFD
95% confidence intervals
Ubiquitin system 3.42e-6
Retinol metabolism 2.73e-5
Biosynthesis of siderophore group nonribosomal pep... 2.72e-4
Steroid hormone biosynthesis 3.25e-4
Metabolism of xenobiotics by cytochrome P450 7.79e-4
Drug metabolism - cytochrome P450 8.55e-4
Glycan biosynthesis and metabolism 1.53e-3
Xylene degradation 1.66e-3
Electron transfer carriers 1.88e-3
Fluorobenzoate degradation 1.94e-3
Protein digestion and absorption 2.34e-3
Systemic lupus erythematosus 2.55e-3
Steroid biosynthesis 2.64e-3
Circadian rhythm - plant 2.64e-3
Fatty acid elongation in mitochondria 2.65e-3
Caffeine metabolism 2.73e-3
Dioxin degradation 3.02e-3
Amyotrophic lateral sclerosis (ALS) 3.28e-3
Carbohydrate digestion and absorption 3.31e-3
Adipocytokine signaling pathway 4.62e-3
African trypanosomiasis 4.88e-3
Carotenoid biosynthesis 5.06e-3
Non-homologous end-joining 5.58e-3
Chagas disease (American trypanosomiasis) 5.73e-3
Penicillin and cephalosporin biosynthesis 6.51e-3
Mineral absorption 0.031
Mean proportion (%)
Difference in mean proportions (%)
p-value (corrected)
(e)
34M4
HFD
95% confidence intervals
Apoptosis 3.87e-5
Steroid hormone biosynthesis 2.01e-4
PPAR signaling pathway 2.02e-4
Linoleic acid metabolism 4.86e-4
Prostate cancer 9.32e-4
Biotin metabolism 1.73e-3
Adipocytokine signaling pathway 1.81e-3
beta-Lactam resistance 2.29e-3
Meiosis - yeast 2.47e-3
Toluene degradation 2.78e-3
Protein digestion and absorption 2.87e-3
Penicillin and cephalosporin biosynthesis 3.40e-3
Pores ion channels 3.68e-3
Type I diabetes mellitus 4.09e-3
Proximal tubule bicarbonate reclamation 7.26e-3
Lipoic acid metabolism 0.014
Flavonoid biosynthesis 0.015
Ubiquinone and other terpenoid-quinone biosynthesis 0.015
Lipopolysaccharide biosynthesis 0.016
"Stilbenoid, diarylheptanoid and gingerol biosynth... 0.016
Phosphonate and phosphinate metabolism 0.017
Pertussis 0.017
Chagas disease (American trypanosomiasis) 0.024
Lipopolysaccharide biosynthesis proteins 0.029
MAPK signaling pathway - yeast 0.035
Mineral absorption 0.039
D-Arginine and D-ornithine metabolism 0.042
African trypanosomiasis 0.044
Mean proportion (%)
Difference in mean proportions (%)
p-value (corrected)
(f)
9M10
HFD
95% confidence intervals
Ubiquitin system 3.65e-5
Retinol metabolism 1.64e-4
Metabolism of xenobiotics by cytochrome P450 5.42e-4
Amyotrophic lateral sclerosis (ALS) 8.21e-4
African trypanosomiasis 1.68e-3
Chagas disease (American trypanosomiasis) 1.91e-3
Penicillin and cephalosporin biosynthesis 1.91e-3
Biosynthesis of siderophore group nonribosomal pep... 2.05e-3
Drug metabolism - cytochrome P450 2.95e-3
Protein digestion and absorption 8.50e-3
Proximal tubule bicarbonate reclamation 0.010
Steroid hormone biosynthesis 0.010
Flavone and flavonol biosynthesis 0.011
Arachidonic acid metabolism 0.015
Geraniol degradation 0.017
Mineral absorption 0.019
Non-homologous end-joining 0.023
Carotenoid biosynthesis 0.024
Steroid biosynthesis 0.025
Fatty acid elongation in mitochondria 0.025
Systemic lupus erythematosus 0.025
Circadian rhythm - plant 0.025
Caffeine metabolism 0.025
Fluorobenzoate degradation 0.026
Meiosis - yeast 0.036
Caprolactam degradation 0.047
Mean proportion (%)
Difference in mean proportions (%)
p-value (corrected)

Figure S3. Prediction of gut microbiota function in T2D mice after *Bifidobacterium* intervention. (a) *B. adolescentis* 26M1; (b) *B. adolescentis* 41M3; (c) *B. adolescentis* 2016; (d) *B. bifidum* 26M7 (e) *B. adolescentis* 34M4; (f) *B. bifidum* 9M10. All results were compared with T2D Model group.

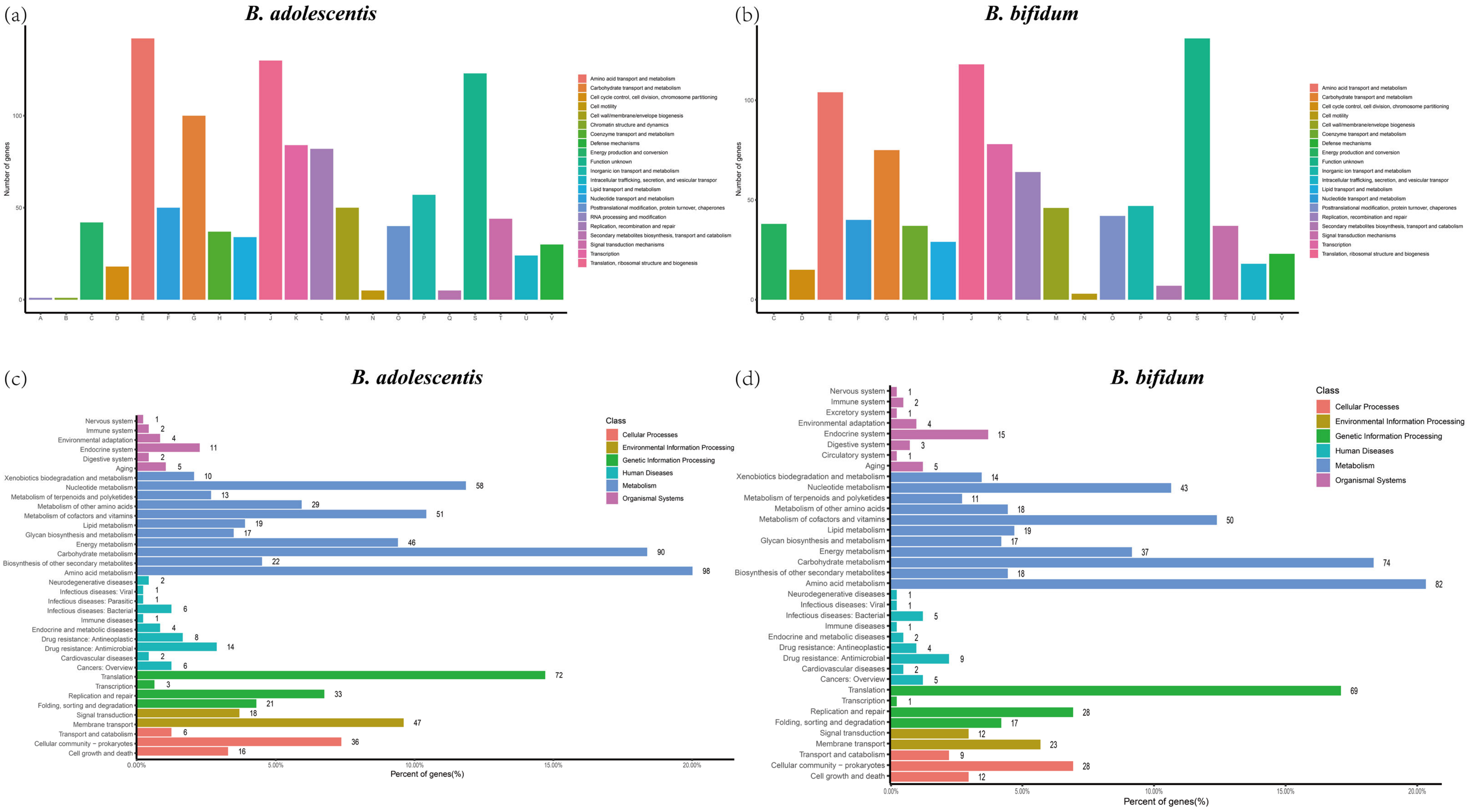

Figure S4. Functional clustering of core genes of *B. adolescentis* and *B. bifidum*. (a, b) Based on COG database; (c-d)Basd on KEGG pathway database.
